# Effects of salt stress on growth and physiological characteristics of *Chamerion angustifolium* seedlings

**DOI:** 10.3389/fpls.2025.1727650

**Published:** 2025-12-10

**Authors:** Caiwei Zhang, Xiaojuan Liu

**Affiliations:** College of Forestry, Gansu Agricultural University, Lanzhou, China

**Keywords:** *Chamerion angustifolium*, salt stress, seedling growth, physiological characters, stress time

## Abstract

**Background:**

Soil salinity is a major abiotic stressor that inhibits plant growth. Assessing the salt tolerance of Chamerion angustifolium (L.) Holub is crucial for understanding its potential in saline environments.

**Methods:**

In this study, *C. angustifolium* seedlings were grown in pots in soil culture, and three concentration gradients of NaCl (50 mmol∙L^-1^, 100 mmol∙L^-1^, 150 mmol∙L^-1^) were set to correspond to mild, moderate and heavy salt stress, respectively; the stress time of 5 d, 10 d and 15 d were set to correspond to short, medium and long time stress. With soil water content of 90%±5% and salt concentration of 0 mmol∙L^-1^ as control, we carried out salt stress treatments to determine the changes of morphological and physiological indices in *C. angustifolium* seedlings under different salt stress levels and different stress times, and to explore the tolerance of *C. angustifolium* seedlings to salt stress.

**Results:**

(1) NaCl stress significantly inhibited the morphological growth of *C. angustifolium* seedlings (P < 0.01). For instance, at 150 mmol·L^-1^ NaCl for 15 days, plant height and leaf area were reduced by approximately 89% and 79%, respectively, compared to the control. Concurrently, osmoregulatory substances such as proline increased markedly, reaching up to 34.7-fold of the control level under severe stress, while antioxidant enzyme activities (e.g., superoxide dismutase, peroxidase) also rose significantly, by up to 393% and 133%, respectively. In contrast, chlorophyll content and leaf relative water content decreased substantially, with total chlorophyll declining by over 50% under the highest salt concentration. With prolonged stress duration, the morphological indices of *C. angustifolium* exhibited a decreasing trend under mild, moderate and severe NaCl stress, a 79% decrease in leaf width under severe stress after 15 days. Meanwhile, the content of osmoregulatory substances and the activity of antioxidant enzymes increased continuously over time across all NaCl concentrations, while chlorophyll content and relative water content consistently declined. (2) Principal component analysis identified malondialdehyde, chlorophyll a, chlorophyll b, and proline as key indicators for screening and evaluating salt tolerance in *C. angustifolium* seedlings.

**Conclusion:**

*C. angustifolium* can be classified as a species with moderate salt tolerance, capable of surviving short-term exposure to 100 mmol·L^-1^ NaCl but at the cost of substantially reduced growth.

## 1 Introduction

Soil salinity represents a major environmental constraint that severely limits the selection of suitable plant species for landscaping and urban greening, particularly in the arid and semi-arid regions of Northwest China (Agastian et al., 2000).

The primary insult of salt stress is osmotic stress, which immediately reduces soil water potential, hindering water uptake and leading to cellular dehydration (Liang et al., 2018). Subsequently, the excessive accumulation of sodium (Na^+^) and chloride (Cl^−^) ions within cells disrupts ionic homeostasis, competing with essential nutrients and inhibiting fundamental enzymatic activities (Ma et al., 2025). At the cellular level, these initial stresses trigger a cascade of physio-biochemical modulations. The impairment of photosynthetic apparatus is not only due to stomatal closure but also involves non-stomatal limitations, including the degradation of chlorophyll pigments and disruption of electron transport chains in chloroplasts (Li et al., 2022). Furthermore, the electron leakage in over-reduced photosynthetic and respiratory chains leads to the burst of reactive oxygen species (ROS), such as superoxide anions and hydrogen peroxide, causing oxidative damage to lipids, proteins, and DNA. These perturbations collectively inhibit growth, disrupt photosynthetic performance, and impair water and nutrient homeostasis (Hasanuzzaman et al., 2012). Crucially, high salt levels profoundly disrupt nutrient uptake patterns and homeostasis by antagonizing the availability and transport of key macronutrients. For instance, Na^+^ competitively inhibits the uptake of potassium (K^+^), a vital cation for enzymatic activation and osmotic adjustment, while also interfering with calcium (Ca²^+^) signaling and nitrate (NO_3_^−^) acquisition (Altaf et al., 2024). To mitigate these adverse effects, plants activate a sophisticated suite of physiological and biochemical responses. These include the compartmentalization of toxic ions into vacuoles, the synthesis of compatible solutes (e.g., proline, glycine betaine) for osmotic adjustment, and the upregulation of the antioxidant enzyme system (e.g., superoxide dismutase, peroxidase, catalase) to scavenge ROS (Wang et al., 2025; Ciriello et al., 2022). Despite these general responses being well-characterized in many species, they are often species-specific, necessitating individual evaluation for plants of horticultural interest.

*Chamerion angustifolium* (L.) Holub, a perennial herb widely distributed across northern temperate zones including Northwest China (Wu and Hong, 2001), has attracted increasing attention for its ornamental value and ecological adaptability (Quan et al., 2010). Its well-formed plant architecture, prolonged flowering period, and pronounced stress resilience make it a promising candidate for landscape use in challenging environments. Previous research has largely focused on the species’ introduction, domestication, and general horticultural traits (Zhai et al., 2009; Quan et al., 2011; Lin et al., 2018; Xu, 2003; Lv, 2004; He and He, 2011; Lei, 2003). Emerging evidence has begun to characterize its salt stress responses, indicating that its seedlings activate antioxidant enzymes (SOD, POD, and CAT) under saline conditions and that seed germination is significantly inhibited at NaCl concentrations exceeding 150 mmol·L^−1^ (Xu, 2023). Furthermore, studies on its subspecies, *C. angustifolium* subsp. *circumvagum*, suggest a certain level of salt tolerance but also reveal impaired photosynthetic function and complex shifts in physiological parameters under high salinity (Zhang, 2024). Another subspecies, *C. angustifolium* subsp. *angustifolium*, is widely recognized for its role as a pioneer species in post-fire ecosystems (Liu et al., 2022). However, these studies are fragmented, and a systematic analysis quantifying the growth and physiological thresholds of the main species under a continuous gradient of salt stress is still lacking. This knowledge gap hinders the targeted utilization of *C. angustifolium* in salt-affected landscaping projects.

Therefore, the present study took the sown seedlings of *C. angustifolium* as the research object, and analyzed the effects of salt stress on the growth indexes and the contents of osmoregulatory substances and antioxidant enzymes in the leaves of *C. angustifolium* seedlings through controlled experiments, expected to clarify its salt tolerance mechanism and threshold, providing a scientific basis for its application in greening projects across saline-affected regions of Northwest China.

## 2 Methods

### 2.1 Experimental material

#### 2.1.1 Seed source and pretreatment

Seeds of *Chamerion angustifolium* were collected from Mapo Township, Yuzhong County, Lanzhou City, Gansu Province, and were subsequently sealed and stored dry at room temperature until use. Before sowing, the seeds were surface-sterilized by soaking in a 20% NaClO solution for 1.5 min, followed by rinsing with distilled water 5–6 times. They were then immersed in water at 40°C for 12 h to promote germination.

#### 2.1.2 Substrate composition and properties

The *C. angustifolium* seedling substrate used was a commercial potting mix, “Strong Seedling No.1” (Gansu Luneng Agricultural Science and Technology Co. Ltd., China). The substrate exhibited a maximum field water holding capacity of 75.52%. Key physicochemical properties were as follows: pH 6.3, total carbon 85.734 mg g^−1^, total nitrogen 6.736 mg g^−1^, total phosphorus 0.797 mg kg^−1^, bulk density of 1.537 g cm^−3^, and water content of 0% (measured by the ring knife method; Institute of Soil Science, Chinese Academy of Sciences, 1978). Prior to use, the substrate was air-dried.

#### 2.1.3 Seedling cultivation and experimental setup

The prepared substrate was packed into pots (15.8 cm in diameter and 13.1 cm in height), with each pot filled with a standardized amount of 400 g. Three holes were made in each pot with 10 seeds per hole at a depth of 0.5 cm. Each treatment was replicated three times (as independent biological replications), with each replication consisting of three pots (technical replicates), resulting in a total of nine pots per treatment and managed with normal watering. After emergence, six seedlings with uniform growth were retained in each pot. Salt stress treatment was initiated one month after sowing, when the plant morphology was essentially stabilized.

### 2.2 Experiment design

The experiment followed a randomized complete block design with two factors: salt stress level (50, 100, and 150 mmol·L^−1^ NaCl, representing mild-S1, moderate-S2, and severe-S3 stress, respectively) and stress duration (5, 10, and 15 days, denoting short-, medium-, and long-term stress). All solutions were formulated using deionized water.

Seventy-two hours prior to salt treatment initiation, a uniform drought preconditioning was applied to all plants to standardize substrate moisture, thereby ensuring uniform penetration and distribution of the subsequent salt solution.

Salt stress was imposed by daily irrigating each pot with 200 mL of the corresponding NaCl solution, prepared with deionized water, applied until drainage occurred from the pot bottom. Any spilled solution was collected in trays and reapplied to its original pot to maintain the intended target salt concentration.

This daily irrigation regime served to uphold a stable salinity environment and to offset water losses from evapotranspiration. The experimental stress period was defined as starting on Day 3, subsequent to the stabilization of substrate salt content (see **Table 1** for the order of irrigation concentrations). Concurrently, systematic plant health monitoring and prophylactic disinfection were carried out to safeguard against biotic confounding factors.

**Table 1:** Treatment of soil salinity.

d/Treatment concentration	50 mmol L^-1^	100 mmol L^-1^	150 mmol L^-1^
1d	200 mL distilled water	200 mL distilled water	200 mL 50 mmol L^-1^ saline solution
2d	200 mL distilled water	200 mL 50 mmol L^-1^ saline solution	200 mL 100 mmol L^-1^ saline solution
3d	200 mL 50 mmol L^-1^ saline solution	200 mL 100 mmol L^-1^ saline solution	200 mL 150 mmol L^-1^ saline solution
Cumulative salt quantity/g	0.29 g	0.58 g	1.75 g
Soil salinity (salinity/air-dried soil weight)/%	0.15%	0.30%	0.45%

### 2.3 Index determination

#### 2.3.1 Growth index

The length of the plant from the base of the plant to the growing point was measured as plant height; the diameter of the plant at the rootstock at ground level was measured as basal diameter; leaf length, leaf width, and leaf area were measured using the ImageJ scientific image-processing software method; growth = nth day measurement - (n-5) day measurement.

#### 2.3.2 Physio-biochemical indices

Chlorophyll (CHL) Content: Chlorophyll was extracted from fresh leaf samples using 80% acetone, and the concentrations of chlorophyll *a* and *b* were quantified spectrophotometrically according to the method of Lichtenthaler (1987).

Malondialdehyde (MDA) Content: The MDA content, indicative of lipid peroxidation, was determined using the thiobarbituric acid (TBA) reaction method as described by Hodges et al. (1999).

Osmoregulatory Substances: Soluble Sugar (Ss) Content was determined using the anthrone-sulfuric acid method following the protocol of Zhang et al. (2006). Soluble Protein (Sp) Content was assayed by the Coomassie Brilliant Blue G-250 staining method, using bovine serum albumin as a standard, as outlined by Bradford (1976).

Proline (Pro) Content was measured by the acid-ninhydrin method following extraction with 3% sulfosalicylic acid, based on the procedure of Bates et al. (1973).

Antioxidant Enzyme Activities: Frozen leaf tissue was homogenized in an ice-cold 50 mM phosphate buffer (pH 7.8) containing 1% (w/v) polyvinylpyrrolidone. The homogenate was centrifuged at 12,000 × g for 20 min at 4°C, and the resultant supernatant was used as the crude enzyme extract for the following assays (Madamanchi et al., 1994).

Superoxide Dismutase (SOD) activity was determined by measuring its capacity to inhibit the photochemical reduction of nitroblue tetrazolium (NBT) at 560 nm, following the methodology of Kamran et al. (2021).

Peroxidase (POD) activity was assayed by monitoring the oxidation of guaiacol at 470 nm, as described by Kamran et al. (2021).

Catalase (CAT) activity was measured by tracking the decomposition of H_2_O_2_ at 240 nm, according to the method of Kamran et al. (2021).

The relative water content (RWC) of *C. angustifolium* leaves was determined by the drying and weighing method (Türk and Erdal, 2015). After weighing 3 g of *C. angustifolium* seedling leaves from each experimental treatment group, the leaves were wiped clean and weighed as fresh weight (*Wf*). After weighing, the leaf materials were immersed in ultrapure water for 4 h. At this time, the *C. angustifolium* leaves were saturated with water, and after removing the leaves, the surface water was sucked dry, and the weight of the *C. angustifolium* leaves at this time was weighed as saturated weight (*Wt*), then pack the weighed, saturated leaves into a dry envelope. Then put the saturated weight of *C. angustifolium* leaves in a dry envelope, put it in a 100-105°C wind drying oven it for 15 min, then lower the temperature of the wind drying oven to 85°C and bake it to a constant weight, then take out the envelope, and when the envelope cooled down to the room temperature, take out the dried *C. angustifolium* leaves, and then weigh the dry weight of the *C. angustifolium* leaves (*Wd*). Reference formula for the calculation of relative water content: $RWC=\frac{\left(Wf-Wd\right)}{\left(Wt-Wd\right)}\times 100\%$.

### 2.4 Data analysis

Microsoft Excel 2010 for data organization, and data analysis was carried out using SPSS 26.1 software. The significance of differences between treatments was tested using Duncan and LSD methods, along with correlation analysis and principal component analysis; Partial Least Squares (PLS-PM) method for the interaction between the growth and physiological indexes of *C. angustifolium* seedlings under salt stress. The pathways of action between the growth and physiological indexes of *C. angustifolium* seedlings under salt stress were plotted using Origin 2021 software.

## 3 Results

### 3.1 Effect of salt stress on the growth of *Chamerion angustifolium* seedlings

**Figure 1:**
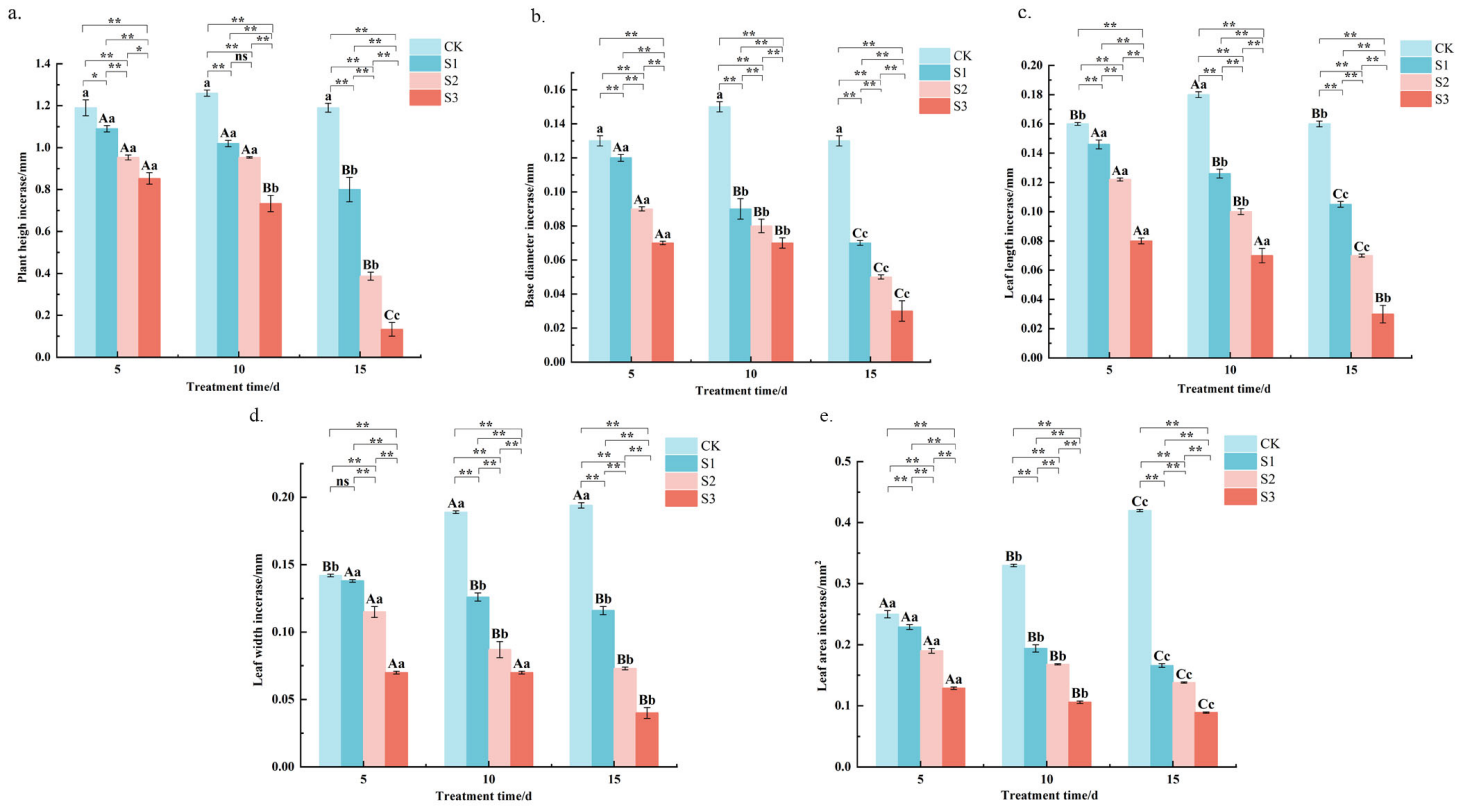
Effects of salt stress on plant height, base diameter, leaf length, leaf width and leaf area growth of *Chamerion angustifolium* seedlings **(a)** Plant height increase; **(b)** Base diameter increase; **(c)** Leaf length increase; **(d)** Leaf width increase; **(e)** Leaf width increase. Different capital letters indicate significant difference between the same salt treatment and different stress times at the 0.01 level; different lowercase letters indicate a significant difference between the same salt treatment and different stress times at the 0.05 level; “**” indicates a significant difference between different salt treatments under the same stress time at the 0.01 level. “*” indicates that there is a significant difference between different salt treatments under the same stress time at the 0.05 level, “ns” indicates that there is no significant difference between different salt treatments under the same stress time, similarly for the following tables.

All salt stress treatments (S1, S2, and S3) significantly suppressed the growth of *Chamerion angustifolium* seedlings compared to the control (*P* < 0.01). For instance, at the end of the stress period, the S3 treatment reduced plant height and total leaf area by approximately 89% and 79%, respectively, relative to the control. The S1 treatment resulted in significantly higher values for all measured growth indices (plant height, basal diameter, leaf length, leaf width, and leaf area) than the S2 and S3 treatments, though these values remained lower than those of the control, with plant height under S1 being 33% lower than the control but 83% higher than under S3 at day 15. The S2 treatment showed intermediate suppression, with values significantly lower than both the control and S1 treatments, basal diameter was intermediate between S1 and S3. The most pronounced growth suppression was observed under the S3 treatment, which consistently yielded the lowest values across all indices at each measurement time (**Figure 1**). Furthermore, a progressive decline in growth was evident with increasing stress duration across all salt treatments. Specifically, the growth rate of plant height under S2 decreased from 0.19 mm d^−1^ during the initial stage to 0.08 mm d^−1^ by the end, indicating a severe inhibition of elongation over time. The results showed that S1, S2, and S3 treatments suppressed the growth rates of plant height, basal diameter, leaf length and width, and leaf area from the early to the end of the stress period.

### 3.2 Effect of salt stress on the relative water content of leaves of *Chamerion angustifolium* seedlings

**Figure 2:**
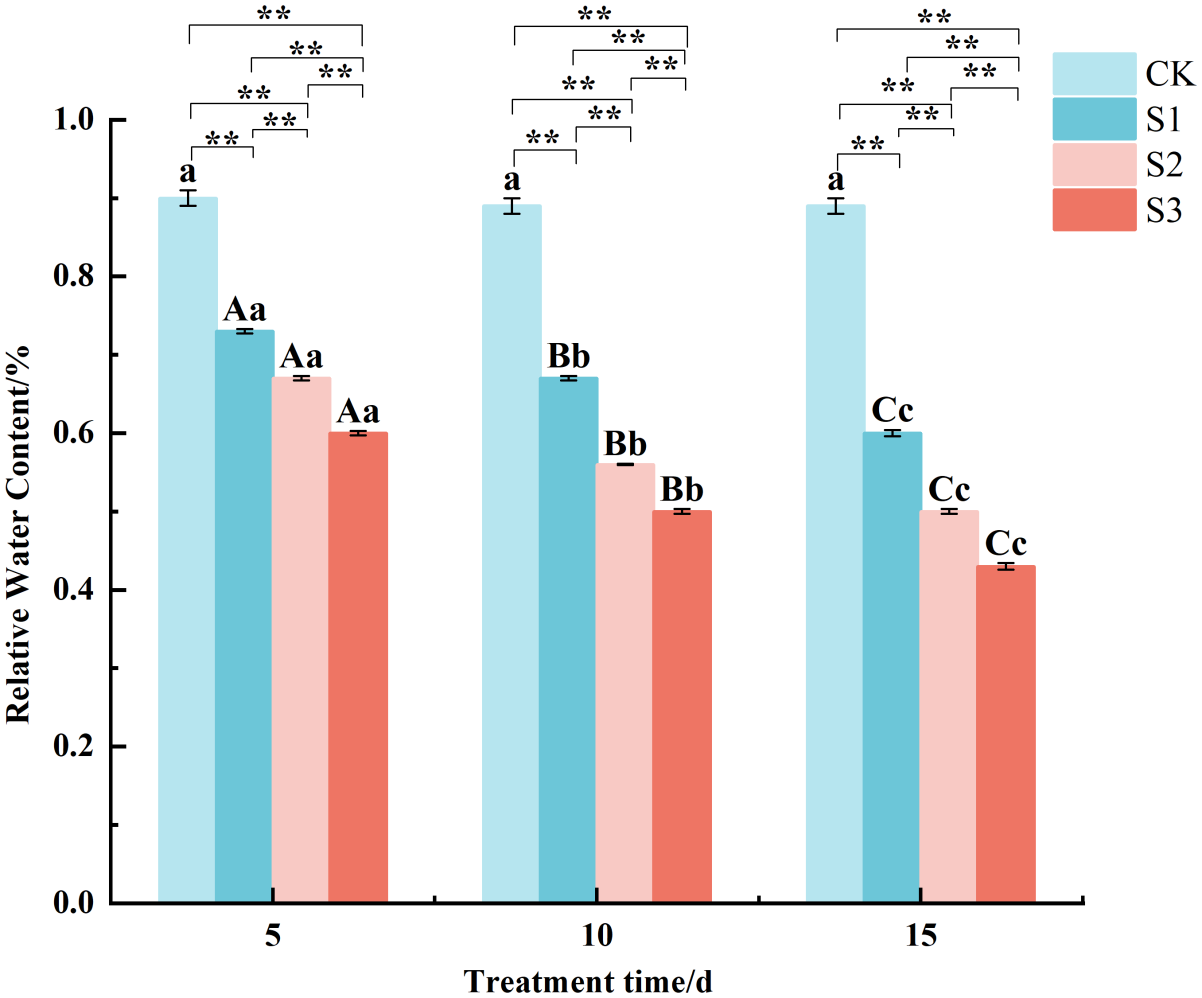
Changes of relative water content in *Chamerion angustifolium* seedlings leaves under salt stress Different capital letters indicate significant difference between the same salt treatment and different stress times at the 0.01 level; different lowercase letters indicate a significant difference between the same salt treatment and different stress times at the 0.05 level; “**” indicates a significant difference between different salt treatments under the same stress time at the 0.01 level. “*” indicates that there is a significant difference between different salt treatments under the same stress time at the 0.05 level, “ns” indicates that there is no significant difference between different salt treatments under the same stress time, similarly for the following tables.

The determination of RWC (**Figure 2**) revealed that RWC was highly significantly lower than the control under all treatments. For example, after 15 days of stress, the RWC in S1, S2, and S3 treatments decreased to approximately 33%, 44%, and 52% of the control value, respectively. All of them showed S1 > S2 > S3 under different stress times, and the differences between treatments were highly significant. The RWC under S3 was consistently 18-28% lower than that of S1 at each measured time point. The RWC of the same treatments all decreased with the increase of stress time. Specifically, in the S2 treatment, RWC dropped from 67% at day 5 to 50% by day 15. The results showed that after being stressed by NaCl, the ability of leaves to retain water decreased continuously with both the duration and degree of stress.

### 3.3 Effect of salt stress on chlorophyll content of leaves of *Chamerion angustifolium* seedlings

**Figure 3:**
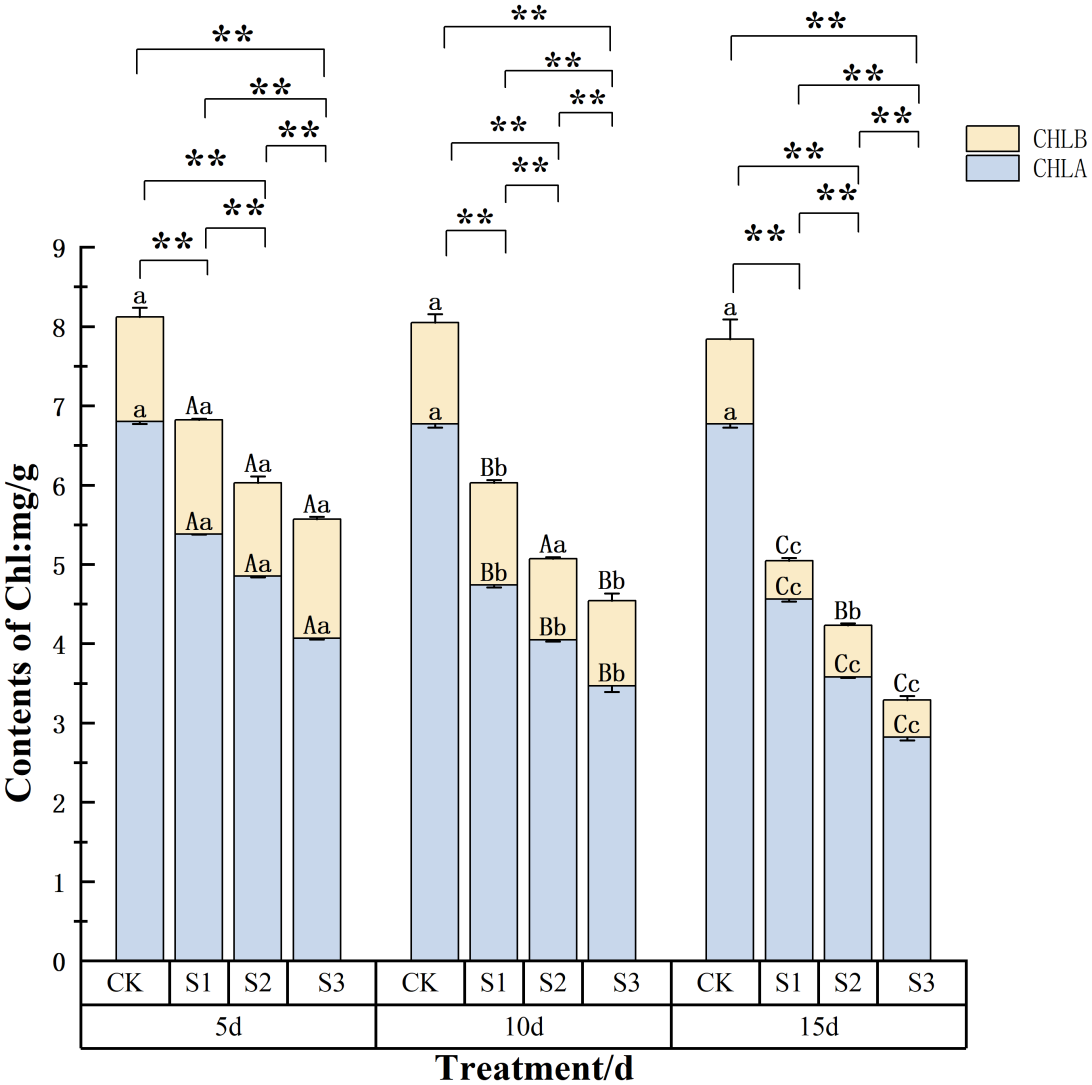
Changes of chlorophyll content in *Chamerion angustifolium* seedlings leaves under salt stress. Different capital letters indicate significant difference between the same salt treatment and different stress times at the 0.01 level; different lowercase letters indicate a significant difference between the same salt treatment and different stress times at the 0.05 level; “**” indicates a significant difference between different salt treatments under the same stress time at the 0.01 level. “*” indicates that there is a significant difference between different salt treatments under the same stress time at the 0.05 level, “ns” indicates that there is no significant difference between different salt treatments under the same stress time, similarly for the following tables.

The CHL*a* and CHL*b* contents of *C. angustifolium* seedling leaves (**Figure 3**) showed CK > S1 > S2 > S3 at different stress times, and the differences among treatments were highly significant. For instance, after 15 days of stress, the total chlorophyll content under the S3 treatment was reduced to only 42% of the control value. With the prolongation of the stress time, the CHL*a* content of all treatments gradually decreased. A clear time-dependent decline was observed in the S2 treatment, where CHL *a* content decreased from 4.9 mg g^−1^ at day 5 to 3.6 mg g^−1^ at day 15. The results showed that after being stressed by NaCl, the total CHL content of leaves decreased continuously with the increase of stress time and stress degree, with the most severe treatment (S3 for 15 days) leading to a 50% reduction compared to the control, indicating that CHL synthesis was severely affected.

### 3.4 Effect of salt stress on the content of MDA and osmoregulatory substances in the leaves of *Chamerion angustifolium* seedlings

**Figure 4:**
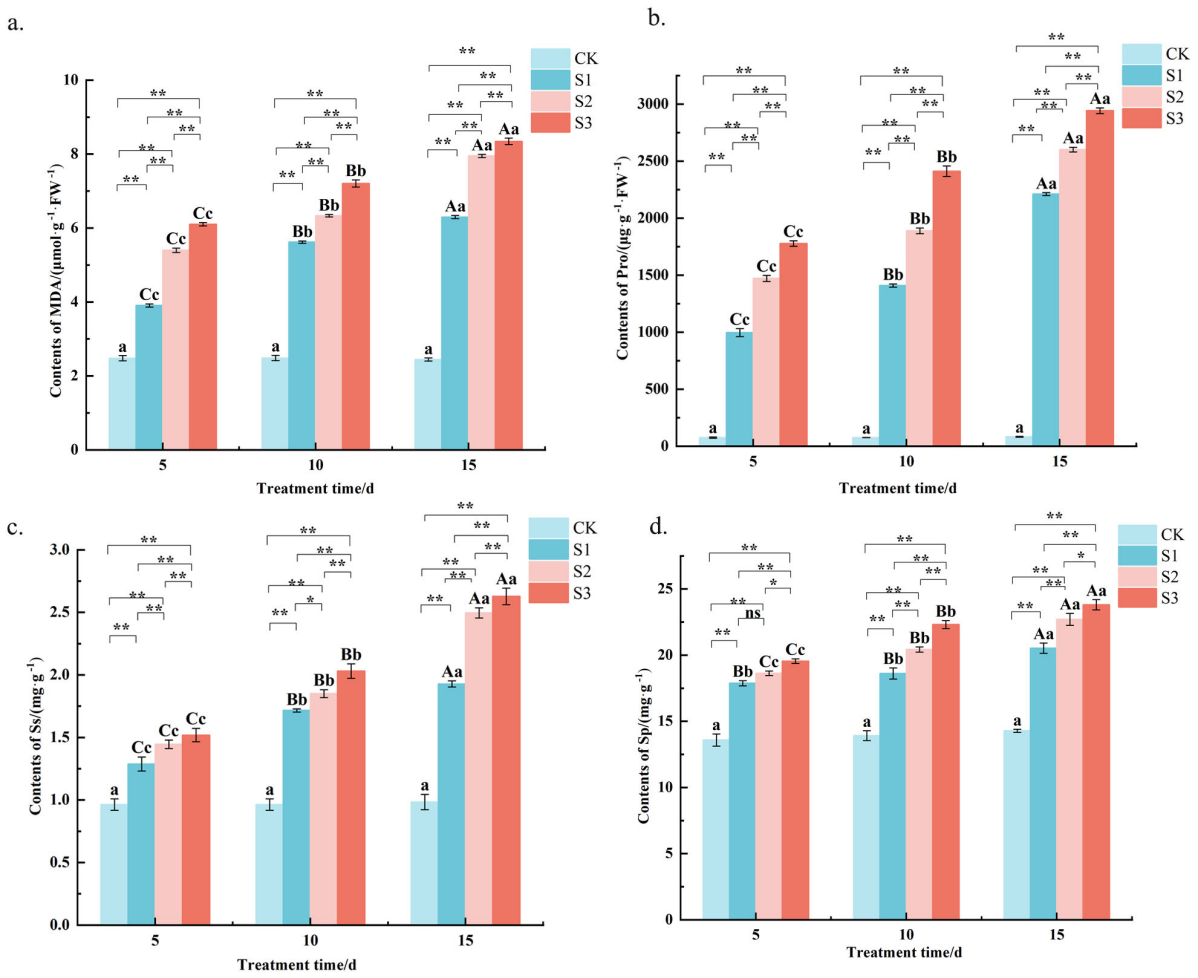
Changes of MDA, Pro, Ss and Sp content in *Chamerion angustifolium* seedlings leaves under salt stress. **(a)** Contents of malondialdehyde; **(b)** Contents of proline; **(c)** Contents of soluble sugar; **(d)** Contents of soluble protein. Different capital letters indicate significant difference between the same salt treatment and different stress times at the 0.01 level; different lowercase letters indicate a significant difference between the same salt treatment and different stress times at the 0.05 level; “**” indicates a significant difference between different salt treatments under the same stress time at the 0.01 level. “*” indicates that there is a significant difference between different salt treatments under the same stress time at the 0.05 level, “ns” indicates that there is no significant difference between different salt treatments under the same stress time, similarly for the following tables.

The determination of MDA content revealed (**Figure 4a**) that after salt stress, the MDA content of *C. angustifolium* leaves was highly significant (*P* < 0.01) higher than that of the control in all treatments (**Table 2**). For example, at the final measurement (15 days), the MDA content under S3 stress reached 8.3 μmol g^−1^ FW^−1^, which was approximately 2.5-fold higher than the control value 2.4 μmol g^−1^ FW^−1^. Under different stress times, the MDA contents showed S3> S2> S1> CK, and the differences between S1 and S2, S3 treatments were highly significant (**Table 2**). The MDA content in S2 was consistently 13-38% higher than in S1 across the time course. With the extension of the stress time, the MDA content of each treatment showed an increasing trend. This temporal increase is exemplified by the S1 treatment, where MDA content rose from 3.9 μmol g^−1^ FW^−1^ at day 5 to 6.3 μmol g^−1^ FW^−1^ by day 15. The results showed that the MDA content of *C. angustifolium* leaves increased under salt stress, and the greater the degree of stress, the higher the MDA content. With the extension of stress time, the MDA content of *C. angustifolium* leaves under mild and moderate, and severe stress gradually increased.

**Table 2:** Two-way ANOVA analysis of effects of salt stress on seedling growth and physiological indexes of *Chamerion angustifolium* seedlings.

Index	Time		Salt		Time×salt	
	SS	F value	SS	F value	SS	F value
Plant height	0.577	236.713**	0.678	278.151**	0.088	36.123**
Base diameter	0.023	1.907**	0.008	0.667**	0.012	1.024**
Leaf length	0.049	2.342**	0.213	10.215**	0.075	3.590*
Leaf width	0.001	24.359**	0.022	912.244**	0.002	63.659**
Leaf area	0.0001	1.844**	0.084	2535.756**	0.009	273.533**
Relative water content	0.043	354.824**	0.255	2086.289**	0.005	39.326**
Chlorophyl A	2.171	550.079**	18.457	4675.574**	0.265	67.008**
Chlorophyll B	1.521	55.546**	0.123	4.492*	0.114	4.166**
Total chlorophyll	7.069	458.907**	21.332	384.734**	0.569	36.906**
Malonaldehyde	44.093	1479.361**	24.729	829.686**	4.992	167.479**
Proline	2339704.280	1416.908**	9086612.55	5502.787**	268124.036	162.374**
Soluble sugar	1.493	981.202**	2.124	1395.998**	0.198	130.273**
Soluble protein	25.634	125.084**	109.554	534.589**	2.300	11.225**
Catalase	1924.702	394.302**	14646.587	3000.56**	2334.581	478.272**
Peroxidase	5304.912	3246.061**	47949.29	29340.039**	2519.379	1541.601**
Superoxide dismutase	110853.215	9553.656**	116372.826	10029.352**	14046.960	1210.608**

SS is sum of squares, **Extremely significant correlation (*P* < 0.01), *Significant correlation (*P* < 0.05).

As shown in (**Figure 4b**), the proline content of each treatment was highly significant (*P* < 0.01) higher than that of the control under different stress times, and all treatments showed S3> S2> S1> CK. For instance, at 15 days, the Pro content in S3-treated leaves reached 2941.9 μg g^−1^ FW^−1^, which was approximately 34.7-fold higher than the control 82.4 μg g^−1^ FW^−1^. The differences between S1 and S2 and S3 treatments were highly significant (**Table 2**). The Pro content in the S2 treatment was consistently 18-48% higher than in S1 across all sampling times. The Pro content under the same treatment all increased with the increase of stress time. This temporal accumulation is clearly demonstrated in the S1 treatment, where Pro content rose from 996.1 μg g^−1^ FW^−1^ at day 5 to 2210.6 μg g^−1^ FW^−1^ at day 15. The results showed that the greater the degree of salt stress, the higher the Pro content, and the longer the stress time, the more the accumulation of Pro.

It was found by the determination of Ss content (**Figure 4c**) that the Ss content of each treatment was highly significant (*P* < 0.01) above the control level at different stress times (**Table 2**). For example, under severe stress (S3) at 15 days, Ss content increased to 2.6 mg g^−1^, representing a 1.6-fold increase compared to the control 1.0 mg g^−1^. Under different stress times, S3> S2> S1> CK was shown among treatments, and the differences between S1 and S2 and S3 treatments were highly significant (**Table 2**). The Ss content in S2 treatment was consistently 8-29% higher than in S1 across the experimental period. With the prolongation of stress time, the Ss’ content under each treatment showed an increasing trend. This temporal pattern was evident in the S1 treatment, where Ss content rose from 1.3 mg g^−1^ at day 5 to 1.9 mg g^−1^ at day 15. The results showed that all concentrations of salt stress treatments promoted the accumulation of Ss in the leaves of *C. angustifolium* seedlings.

The Sp content of *C. angustifolium* seedling leaves under each treatment of salt stress was highly significant (*P* < 0.01) higher than that of the control (**Figure 4d**). For instance, under the S3 treatment at 15 days, Sp content accumulated to 23.8 mg g^−1^, which was about 1.7-fold of the control level 14.3 mg g^−1^. At different stress times, the Sp contents were S3> S2> S1> CK, and the differences between S1, S2 and S3 treatments were significant (*P* < 0.05) or highly significant (*P* < 0.01) (**Table 2**). On average across all time points, the Sp content in S2 was 4-11% higher than that in S1. With the extension of the stress time, the Sp content of each treatment showed an increasing trend. This is exemplified by the S1 treatment, where Sp content increased from 17.9 mg g^−1^ at day 5 to 20.5 mg g^−1^ at day 15. The results showed that the different levels of salt stress treatments promoted the accumulation of Sp.

### 3.5 Effect of salt stress on the content of MDA and osmoregulatory substances in the leaves of *Chamerion angustifolium* seedlings

**Figure 5:**
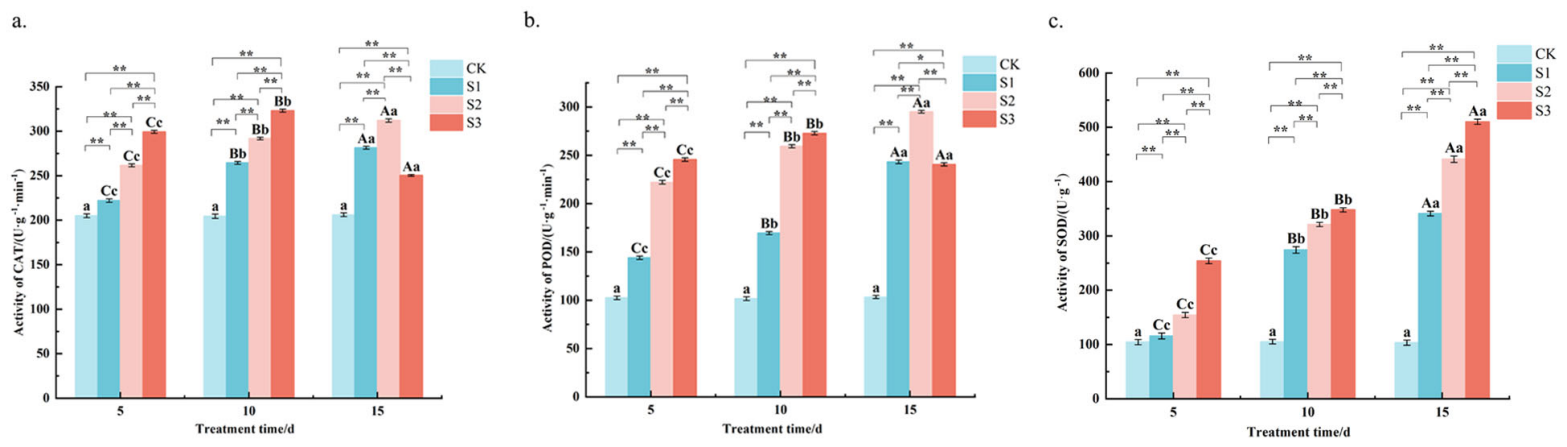
Changes of CAT, POD, SOD content in *Chamerion angustifolium* seedlings leaves under salt stress. **(a)** Activity of catalase; **(b)** Activity of peroxidase; **(c)** Activity of superoxide dismutase. Different capital letters indicate significant difference between the same salt treatment and different stress times at the 0.01 level; different lowercase letters indicate a significant difference between the same salt treatment and different stress times at the 0.05 level; “**” indicates a significant difference between different salt treatments under the same stress time at the 0.01 level. “*” indicates that there is a significant difference between different salt treatments under the same stress time at the 0.05 level, “ns” indicates that there is no significant difference between different salt treatments under the same stress time, similarly for the following tables.

The CAT activity was measured (**Figure 5a**) and showed that at 5 and 10 d of stress, CAT activity was S3> S2> S1> CK, and the difference between S1, S2 and S3 treatments was highly significant (*P* < 0.01). For example, at 10 days, CAT activity under S3 reached 323.1 U g^−1^ min^−1^, which was 0.6-fold higher than the control and 22% higher than the S1 treatment. At 15 d of stress, the S2 treatment was highly significantly higher than the control and the S1 and S3 treatments. Specifically, S2 activity at day 15 was 312.1 U g^−1^ min^−1^ significantly exceeding the S3 value of 250.4 U g^−1^ min^−1^ at the same time point. Except for S3 treatment, where CAT activity increased and then decreased, CAT activity increased with the extension of stress time in all treatments. The results showed that CAT activity under S3 treatment was the highest at different stress times, and CAT activity was promoted under all treatments. The S3 activity peaked at 323.1 U g^−1^ min^−1^ on day 10 before declining to 250.4 U g^−1^ min^−1^ on day 15. The results showed that CAT activity was highest under S3 treatment at different stress times, and CAT activity was promoted under all treatments.

The determination of POD activity (**Figure 5b**) revealed that the POD activity of S1 treatment was highly significant (*P* < 0.01) higher than the control and highly significantly lower than S2 and S3 treatments at different stress times. The POD activity of S2 treatment was highly significant (*P* < 0.01) higher than the control and S1 treatments and lower than S3 treatment at both 5 d and 10 d of stress. At 15 d of stress, S1 was highly significantly higher than the control and S2 and S3 treatments. the S3 treatment was highly significantly higher than the control and the other two treatments at 5 and 10 d of stress, and was highly significantly lower than the S2 treatment at 15 d of stress, but higher than the control and S1 treatments. Over time, POD activity under S1 and S2 treatments showed a gradual increase, with S2 increasing from 222.1 U g^−1^ min^−1^ at day 5 to the aforementioned peak at day 15. In contrast, the POD activity under S3 treatment increased initially but then decreased sharply after 10 days. The results showed that POD activity was promoted under each concentration of stress treatment at different stress times.

The SOD activity was measured (**Figure 5c**) and was found to be S3> S2> S1> CK at different stress times, and the difference between S1, S2 and S3 treatments was highly significant (*P* < 0.01). For instance, at 10 days of stress, the SOD activity under S3 treatment reached 347.9 U g^−1^, which was approximately 2.3-fold higher than the control 105.3 U g^−1^ and 27% higher than the S1 treatment 274.2 U g^−1^. With the extension of the stress time, the SOD activity of each treatment showed an increasing trend. A consistent gradient was observed, where SOD activity in S2 was, on average, 17-33% higher than in S1 across the stress period. The results showed that the SOD activities were promoted under each concentration of stress treatments at different stress times.

### 3.6 Comprehensive analysis and evaluation of salt tolerance in *Chamerion angustifolium* seedlings

#### 3.6.1 Correlation analysis

**Figure 6:**
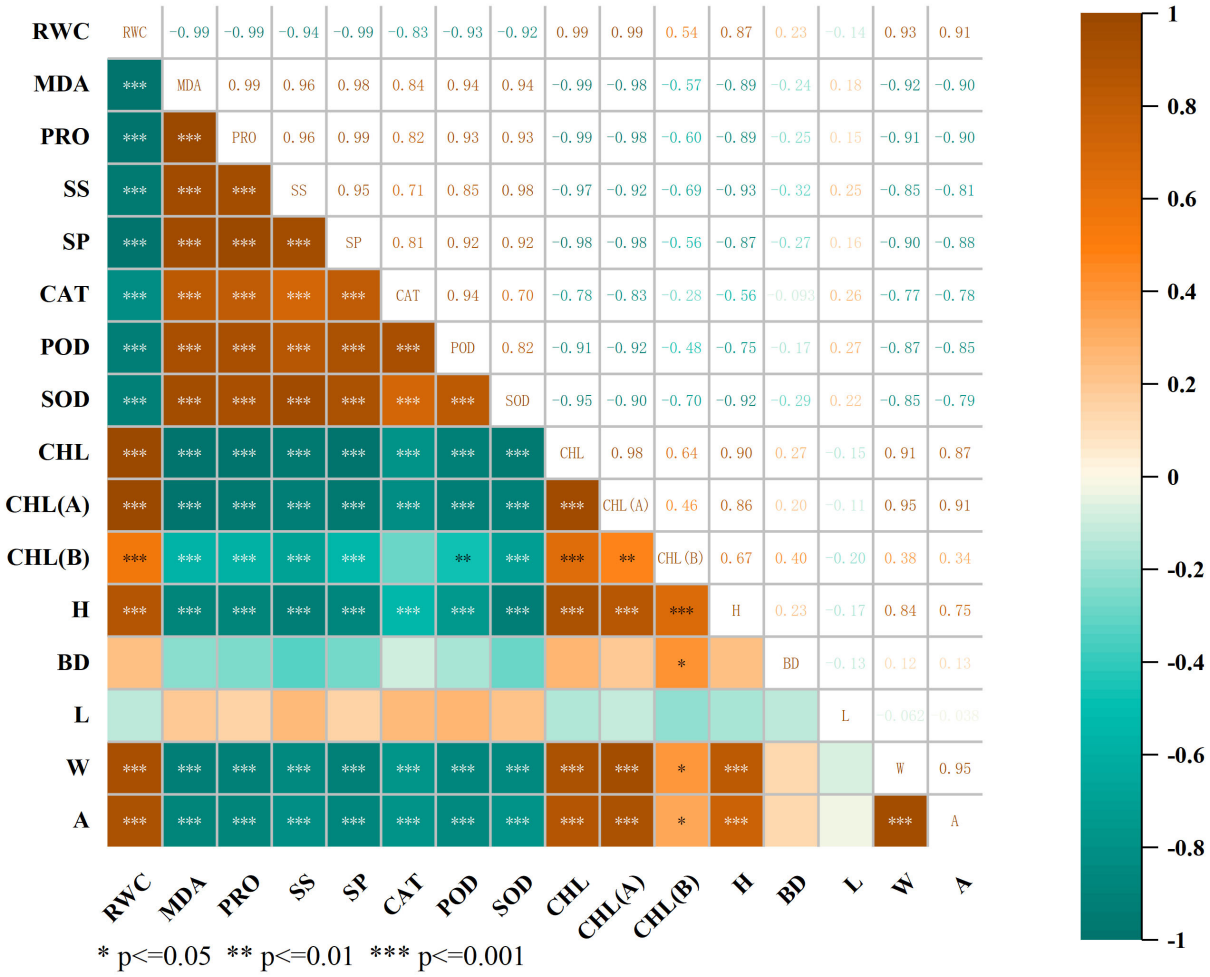
The correlation heat map. MDA: malonaldehyde; PRO: proline; SS: soluble sugar; SP: soluble protein; CAT: catalase; POD: peroxidase; SOD: superoxide dismutase; CHL: total chlorophyll; CHL(A): chlorophyll A; CHL(B): chlorophyll B; H: plant height increase; BD: base diameter increase; L: leaf length increase; W: leaf width increase; A: leaf area increase.

Correlation analysis (**Figure 6**) revealed that there were different degrees of correlations between all morphological and physiological indicators of *C. angustifolium*. There were two-by-two highly significant negative correlations (*P* < 0.01) between RWC and MDA, SP, SS, CAT, POD, SOD, and two-by-two highly significant positive correlations (*P* < 0.01) between RWC and CHL, CHL(A), BD, W, A, H, and two-by-two highly significant positive correlations (*P* < 0.01) between CHL, CHL(A), W, A, and A in *C. angustifolium*. The positive correlation between CHL and CHL(*A*), W, A was highly significant (*P* < 0.01); the positive correlation between CHL and CHL(*A*), W, A in *C. angustifolium* was highly significant (*P* < 0.01).

#### 3.6.2 Principal component analysis

**Figure 7:**
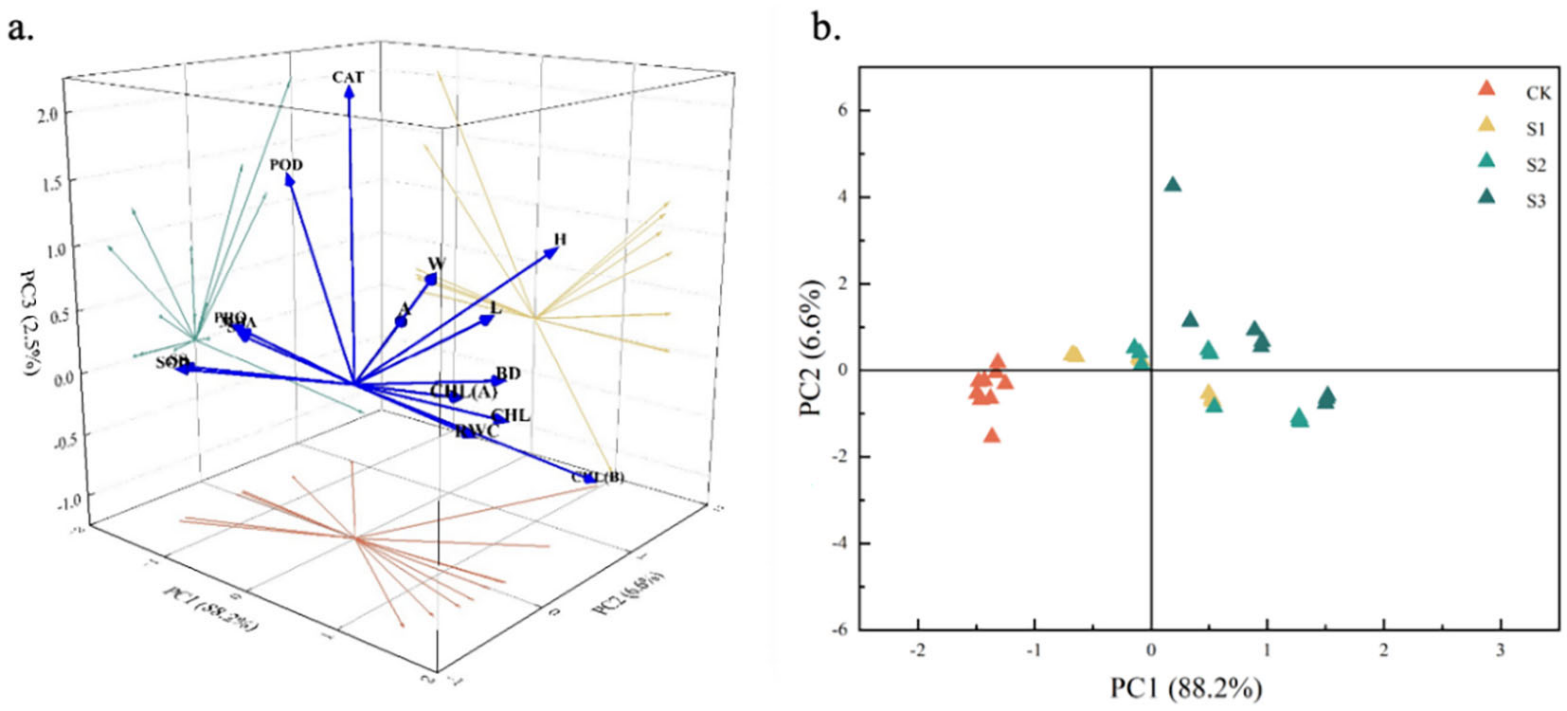
Principal component score map of physiological and morphological indexes of Chamerion angustifolium seedlings. **(a)** The first two principal component loads of indexes; **(b)** Scatter plots of samples based on PC1 and PC2.

Based on the above correlation analysis results, this study further applied principal component analysis to downscale the data (**Figure 7**). Two principal component factors with a cumulative variance contribution rate greater than 80% and an eigenvalue greater than 1 were screened out (**Table 3**), with a cumulative contribution rate of 94.813%, corresponding to larger eigenvectors of MDA, CHL, PRO, and CHL(B), which mainly reflected the malondialdehyde content, proline content, chlorophyll, and chlorophyll b characteristics, suggesting that these four indexes can be used as important indicators for the screening and evaluation of salt resistance of *C. angustifolium* seedlings. The results showed that these four indicators can be used as important indicators for screening and evaluation of salt resistance of *C. angustifolium* seedlings.

**Table 3:** Principal component analysis results of the physiological response of *Chamerion angustifolium* growth under salt stress.

Index	The first principal component	The second principal component
RWC	0.264	-0.075
MDA	-0.265	0.030
PRO	-0.265	0.001
SS	-0.257	-0.196
SP	-0.263	0.020
CAT	-0.219	0.394
POD	-0.248	0.196
SOD	-0.253	-0.220
CHL	0.265	0.050
CHL(A)	0.262	-0.124
CHL(B)	0.169	0.700
H	0.244	0.286
BD	0.261	0.045
L	0.259	-0.002
W	0.250	-0.199
A	0.239	-0.284
Eigen value	14.112	1.058
Variance contribution/%	88.198	6.615
Cumulative contribution rate/%	88.198	94.813

Scatter plots were constructed according to PC1 and PC2 to show the relationship between the indicators of *Chamerion angustifolium*. It can be seen from **Figure 7b** that different degrees of salt stress had a greater impact on the growth and physiology of *C. angustifolium*, while the distribution range of the sample plants on PC1 was smaller than that of PC2. The distribution of the sample plants of CK was more centralized in the scatter plots, and the distribution of the scatter points of the other treatments was more dispersed.

#### 3.6.3 Two-factor analysis

The results of two-way ANOVA showed (**Table 2**) that NaCl stress had highly significant (*P* < 0.01) effects on the growth of plant height, basal diameter, leaf length, leaf width and leaf area of *C. angustifolium* seedlings. The values of the effects of the degree of salinity stress and the time of stress on the growth amount of plant height of *C. angustifolium* seedlings were salinity > time > time × salinity, the effects on the growth amount of basal diameter were time > time × salinity > salinity, and the effects on the growth amount of leaf length, leaf width, and leaf area were salinity > time × salinity > time. Therefore, salinity was the main factor affecting the amount of growth in height, basal diameter, leaf length, leaf width and leaf area of *C. angustifolium* seedlings.

NaCl stress had an extremely significant effect on the RWC of leaves of *C. angustifolium* seedlings (*P* < 0.01). The influence values of salt stress degree and stress time on the RWC of *C. angustifolium* seedlings were salt > time > time × salt. Therefore, salt content is the main factor affecting the RWC of the leaves of *C. angustifolium* seedlings.

NaCl stress had highly significant effects (*P* < 0.01) on total CHL, CHL*a* and CHL*b* contents in *C. angustifolium* seedlings, and the values of salt stress degree and stress time on CHL*a* and total CHL contents in *C. angustifolium* seedlings were salt > time > time × salt, and the values of time > salt > time × salt for CHL*b*. Therefore, salinity was the main factor affecting CHL*a* and total CHL content in *C. angustifolium* seedlings, and time was the main factor affecting CHL*b* content in *C. angustifolium* seedlings.

NaCl stress had highly significant effects (*P* < 0.01) on MDA content, Pro content, Ss content and Sp content in *C. angustifolium* seedlings, and the effect values of the degree of salt stress and time of stress on MDA content in *C. angustifolium* seedlings were time > salt > time × salt. The effect values of the degree of salt stress and time of stress on Ss content, Pro content and Sp content in *C. angustifolium* seedlings were salt > time > time × salinity. Therefore, time was the main factor affecting MDA content and salinity was the main factor affecting Ss content, Pro content and Sp content in *C. angustifolium* seedlings.

NaCl stress had a highly significant (*P* < 0.01) effect on the antioxidant enzyme activities of both *C. angustifolium* seedlings. From the F-value of the degree of salt stress and the time of stress, the degree of effect on CAT activity was all as salt > time × salt > time, and the degree of effect on POD activity and SOD activity was salt > time > time × salt. Therefore, salinity was the main factor affecting CAT activity, POD activity and SOD activity in *C. angustifolium* seedlings.

#### 3.6.4 Partial least squares - path model

**Figure 8:**
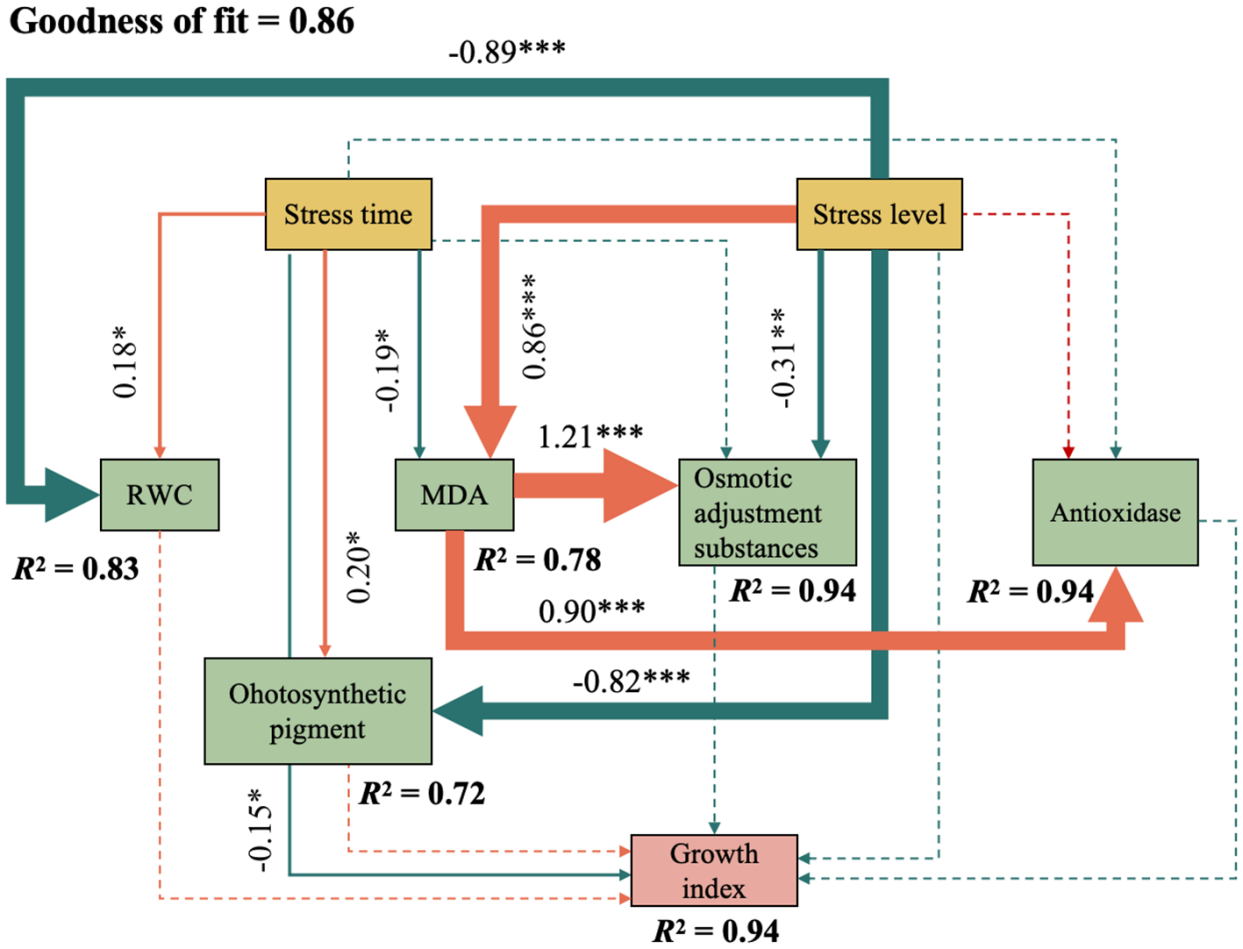
A partial least square model of the physiological and growth influence mechanism of *Chamerion angustifolium*. The goodness of fit = 0.86. *indicates P < 0.05, **indicates P < 0.01, ***indicates P < 0.001. In the inner model. The orange and green arrows indicate positive correlation and negative correlation, respectively; the solid line indicates a significant relationship between the two indexes; the dashed line indicates that the relationship between the two indexes is not significant. The number on the arrow line and the thickness of the arrow line represent the path coefficient value; the R^2^ indicates the explanatory degree of the model.

Partial least squares-path modeling (PLS-PM) was used to analyze the mechanism of physiological indicators of *C. angustifolium* on growth under salt stress (**Figure 8**). The model fit was Gof=0.86, indicating that the overall prediction accuracy of the model was high.

The path coefficients (**Figure 8**) reveal the specific physiological pathways impacted by salt stress. Most critically, the degree of stress exerted a strong direct negative effect on growth (path coefficient: -0.89, *p* < 0.001). Furthermore, it initiated a cascade of physiological disruptions: a significant positive effect on MDA content (indicating severe membrane lipid peroxidation and oxidative damage) and a significant negative effect on relative water content (RWC) (reflecting osmotic stress-induced dehydration) and photosynthetic pigments (impairing photosynthetic capacity). RWC, MDA, osmoregulatory substances, antioxidant enzyme activity, and photosynthetic pigment content together explained 94% of the variance of the growth indicators in seedlings.

## 4 Discussion

The detrimental impact of soil salinity on plant growth and development is well-documented, primarily through induction of ionic imbalance, osmotic stress, and secondary oxidative damage (Zhou et al., 2015). Our findings corroborate this overarching theme, demonstrating that NaCl stress significantly inhibited the morphogenesis of *Chamerion angustifolium* seedlings. However, a temporal analysis reveals that the plant’s response to salt stress is not static, but a dynamic process with distinct early and late phases, the manifestation of which varied across physiological attributes.

High salt environments significantly inhibit plant morphogenesis, as evidenced by limited plant height, impeded stem thickening, and suppressed leaf expansion (Yi et al., 2021). Salt stress inhibits elongation of plant leaves and main stems, resulting in reduced leaf area index and weak, low-growth plants (Hong et al., 2020). Our results showed that growth inhibition was an immediate response, detectable from the first measurement at 5 days. This initial suppression is primarily attributed to osmotic stress, which rapidly compromises water uptake (Zhang et al., 2021). However, a deeper analysis establishing linkages between these attributes across varying salt concentrations and durations reveals a coherent narrative of progressive physiological disruption. The primary linkage here lies in the ionic and osmotic stress. High external salt concentration leads to Na^+^ accumulation and K^+^ depletion within plant cells, disrupting enzymatic functions and membrane integrity. Consequently, the plant’s ability to absorb water and essential nutrients is compromised. Therefore, the observed concurrent reduction in plant height (stem cell elongation), basal diameter (cambial activity), and leaf area (leaf cell expansion) are not independent events but are interconnected manifestations of the same initial cause: disrupted cellular homeostasis (Su et al., 2016). The results of Liu et al. (2017) showed that both root and aboveground growth and development of *Chenopodium quinoa* Willd. seedlings exhibited a significant concentration-dependent inhibitory effect in NaCl-stressed environments. As the gradient of salt treatment concentration increased and the prolongation of the stress time, the degree of growth restriction of each organ of the plant showed a trend of gradual increase, which was similar to the results of this study.

Changes in relative water content can visualize the efficiency of plants in regulating water metabolism under environmental stress conditions, which is an important physiological parameter for evaluating drought resistance of plants. In this study, the relative water content showed a decreasing trend with both the increase of NaCl solution concentration and the extension of stress time, indicating that the high salt environment increases the osmotic pressure of the soil solution, which makes it difficult for the plant root system to absorb water, leading to a decrease in the relative water content of the leaves (Xiong et al., 2025). Leaf dry matter is all the material remaining in the leaf after the removal of water, and these components work together to maintain plant life activities and growth and development (Liu, 2006). With prolonged stress, the growth inhibition intensified, transitioning from an initial osmotic limitation to a state of chronic energy deficit and accumulated damage, as reflected in the continuous reduction of growth rates and the eventual accumulation of leaf dry matter—a potential consequence of growth dilution and the accumulation of protective compounds (Ding et al., 2022).

Chlorophyll can directly reflect the photosynthetic metabolic capacity of leaves (Liao et al., 2010). In the initial phase of stress, chlorophyll content began to decline, consistent with findings in *Amorpha fruticosa* and cotton (Wang et al., 2021; A· Maimaiti et al., 2017). However, the most pronounced degradation occurred in the late phase under sustained, severe stress. This progressive, time-dependent decrease signifies a shift from initial stress signaling to irreversible damage to the photosynthetic apparatus, likely exacerbated by the cumulative effects of ion toxicity and oxidative stress (Ma et al., 2015), ultimately leading to a severe limitation on the plant’s carbon and energy supply, as seen in our study and others (Wang et al., 2013).

When plants are subjected to salt stress, a significant increase in intracellular reactive oxygen species levels triggers a chain reaction of lipid peroxidation (Xie et al., 2012), which ultimately leads to the impairment of the structural and functional integrity of the plasma membrane system (Mi et al., 2018). The concentration of MDA, a key marker for this oxidative damage, showed a continuous, time-dependent increase throughout the experiment (Zhu et al., 2022; Ma et al., 2018). This indicates that the initial oxidative burst (early phase) evolved into sustained and escalating membrane damage in the late phase. The gradual elevation of MDA content with both increasing concentration and duration underscores that the membrane structure suffered progressive and severe damage (Chen et al., 2019), moving beyond initial perturbation to a state of chronic integrity loss.

Under high-salt environments, plants face multiple stresses simultaneously, including oxidative stress caused by oxygen radicals as well as osmotic stress (Wu, 2015). The synergistic accumulation of osmoregulatory substances can effectively reduce the cellular water potential, thus alleviating physiological water deficit caused by adversities such as drought or salinity (Liu et al., 2011). Our results showed an immediate initiation of this response, with osmoregulant levels rising significantly by day 5. Critically, this accumulation was not a transient event but was sustained and even enhanced over time, a pattern also observed in *Agropyron mongolicum* (Yu et al., 2021). This persistent effort highlights osmotic adjustment as a long-term strategy in *C. angustifolium*; however, the substantial resource allocation required for this sustained synthesis likely contributed to the observed growth suppression in the late phase (Hu et al., 2019).

In high-salt environments, intracellular metabolic homeostasis in plant leaves is disrupted, triggering severe oxidative stress. This oxidative stress impairs the structural and functional integrity of the biofilm system. As a coping mechanism, a well-established defense system of antioxidant enzymes is activated in plants, in which SOD, POD, and CAT play key roles (Li et al., 2017). In the early to mid-phase of stress, we observed a general upregulation of these enzymes, consistent with their role in initial defense (Ren et al., 2022). However, in the present research, while SOD activity in *C. angustifolium* remained elevated, presumably to handle ongoing superoxide production, the activities of POD and CAT under severe stress (S3) exhibited a marked decline by day 15. Su et al. (2022) found that during salt stress, when the NaCl concentration exceeded the plant’s own regulatory threshold, both its metabolic homeostasis and antioxidant defense system were dysfunctional, leading to failure of the protective mechanism and ultimately inhibiting the normal growth and development of *Viola tricolor* L. seedlings. This may stem from the fact that the large amount of Na^+^ ions in the inward flow may trigger the intensification of membrane lipid peroxidation, which destroys the integrity of the cell membrane structure, and then interferes with the normal physiological and metabolic processes, and ultimately leads to the reduction of POD activity. Under the conditions of short-term salt treatment at low concentrations, antioxidant enzymes such as POD can effectively scavenge reactive oxygen radicals in seedlings; however, when the salt concentration exceeds a specific critical value, the activity of these enzymes is inhibited, resulting in a significant reduction in the antioxidant defense capacity of the plant, which was similar to the findings of others (Zhuang et al., 2010; Xu et al., 2023).

The results of correlation analysis showed that the relative water content and growth rate of *C. angustifolium* seedlings were negatively correlated with as well as chlorophyll and osmotic regulation, indicating that as the degree of salt stress deepened, the relative water content of the plant declined, resulting in osmotic stress, and the plant produced osmotic regulating substances to alleviate the osmotic stress. At the same time, the chlorophyll synthesis was affected, affecting the photosynthetic rate, thus slowing down the growth rate and reducing the accumulation of dry matter in the plant. After being subjected to stress the plant’s cell membranes are destroyed by a large amount of reactive oxygen species, and under short-term stress, *C. angustifolium* activates the protective enzyme system and osmoregulatory mechanisms to ensure normal physiological activities. Under long-term stress and severe stress, the physiological growth of *C. angustifolium* will be seriously impaired when it exceeds the range of its self-regulation ability.

These findings are further refined and substantiated by our principal component analysis (PCA). The PCA conclusively identified malondialdehyde (MDA), proline (PRO), total chlorophyll (CHL), and chlorophyll b (CHL*b*) as the four most critical indicators for evaluating salt tolerance. This selection is physiologically sound: MDA serves as a key marker for oxidative damage caused by reactive oxygen species, PRO represents the core osmotic adjustment mechanism, while the decline in CHL and CHL*b* directly reflects the damage to the photosynthetic apparatus. Under short-term stress, the activation of protective enzyme and osmoregulatory systems can mitigate this damage. However, as evidenced by the PCA loadings and their correlations with plant performance, when stress becomes prolonged or severe—exceeding the plant’s self-regulatory capacity—the cumulative damage to membranes (indicated by MDA) and the photosynthetic system (indicated by CHL/CHL*b*) becomes critical, leading to the severe physiological impairment we observed.

Notably, the path model quantified how these physiological responses mediated the growth inhibition. The negative path from RWC to growth and the positive path from osmoregulatory substances to growth suggest that water deficit and the consequent osmotic adjustment effort are key processes influencing final biomass. While antioxidant enzyme activity was upregulated by stress (positive path), its non-significant path to growth implies that this protective response alone was insufficient to prevent oxidative damage from curtailing growth under prolonged or severe stress.

Collectively, the model attributes 94% of the variance in growth indicators (R² = 0.94) to the integrated effects of these physiological variables. The PLS-PM therefore robustly supports the conclusion that salt stress suppresses the growth of *C. angustifolium* primarily through a combination of direct inhibitory effects and indirect effects mediated by osmotic stress, oxidative membrane damage, and photosynthetic decline.

## 5 Conclusion

In conclusion, this study demonstrates that the physiological and growth responses of *Chamerion angustifolium* to salt stress are determined by the integrated effects of both stress intensity and duration. While the seedlings activated adaptive mechanisms—such as the accumulation of proline and enhanced antioxidant enzyme activities—these responses were insufficient to prevent significant growth inhibition even at 50 mmol·L^−1^ NaCl. The detrimental effects, including membrane damage (reflected by MDA accumulation) and chlorophyll degradation, were progressively amplified with both increasing salt concentration and prolonged exposure time. Therefore, *C. angustifolium* can be classified as a species with moderate salt tolerance, capable of surviving short-term exposure to 100 mmol·L^−1^ NaCl but at the cost of substantially reduced growth. The PCA and statistical analyses further established that MDA, total chlorophyll, chlorophyll b, and proline are pivotal indicators for screening and evaluating this inherent salt tolerance in *C. angustifolium* seedlings.

It should be noted that this study focused on a single genotype and a uniform soil type to establish the baseline response of *C. angustifolium* to salt stress. Future investigations incorporating diverse ecotypes and different soil properties would be invaluable for understanding the genotype-by-environment interactions that shape the species’ salt tolerance in natural settings.

## Data Availability

The original contributions presented in the study are included in the article/supplementary material. Further inquiries can be directed to the corresponding author.
